# Arsenic for Common Use

**Published:** 1891-07

**Authors:** 


### ARSENIC FOR COMMON USE.

(From the New York *Times*, March 1st.)

The protests of the Massachusetts Homœopathic Medical Society against the use of arsenic in the manufacture of wall paper, textile fabrics, and other articles commonly found in the sleeping and living rooms of dwelling-houses have led the Massachusetts Legislature's Committee on Public Health to take testimony concerning this matter. We referred, on the 8th ult., to the reports laid before the Homœopathic Medical Society by Professor Calder, of Brown University, Dr. Talbot, and others. At the first hearing before the Legislative Committee many interesting statements were made by the complainants, while ex-Governor Long appeared as counsel representing certain manufacturers of paper.

Among those who testified was Dr. Wm. P. Bolles, one of the surgeons of the City Hospital, who described at length the experience of his son, a child four years old, who has suffered from arsenical poison for two years. After the cause of his illness had been discovered a search for arsenic was made in the rooms of the house. In the paper borders on the walls of his bed-room arsenic was found, and an examination of several boxes that contained his toys revealed considerable quantities of the same poison in the coverings and linings. More of it was discovered in the plush coverings and the lining of an arm-chair, in the frescoes on the ceiling, and in some scarlet braid attached to his clothing. The dust on the tops of the windows and door frames in the room where arsenic had been used in the frescoes was found to be arsenical. The wall-paper, except the borders, was not poisonous. Owing to the effect of the arsenic the boy's health has suffered serious and probably permanent injury. Dr. Bolles also spoke of one of his patients, a woman who had died of disease caused by the absorption of arsenic, after a distressing illness of several years. The autopsy revealed the presence of arsenic and the irritating effect of the poison. Both in the room where she died and in the room where her illness began the wall-paper was found to be loaded with arsenic. He had discovered arsenic in a woolen wrapper and a dress worn by another patient whom he had treated for arsenical poisoning, and he asserted that in his opinion the unconscious absorption of arsenic from wall-papers and fabrics was the cause of general debility and weakness in many cases.

Another witness was Dr. Charles P. Strong, of the Massachusetts General Hospital, who cited the experience of two patients. One of them had suffered from debility for several years, and during the greater part of that time had lived in two rooms of a certain house. A year ago she came under his care and was treated for arsenical poisoning. She was taken from the house and is beginning to recover. "Arsenic was found in large quantities," says the report of his testimony, "in the draperies of her room and in the lounge in the library. The wall-papers also contained arsenic." Dr. Horace Packard spoke of a person in his own house who had been poisoned. "The wall-paper in the room was covered with a pigment, which was examined and found to contain a large amount of arsenic. He was removed to another house and the illness disappeared."

Thomas J. Gargan told the Committee that on November 27th, he had undergone a surgical operation, and that his failure to recover from the effect of it had caused his physician to suspect that he had absorbed arsenic. There was no paper on the wall of his room, but an examination of two "comforters" used on the bed disclosed large quantities of the poison in the coloring matter. Arsenic was also found in the coloring of the cover of a lounge and in the colors on the cornice of the wall. He was still suffering from the poison. Ernest F. Henderson testified that, while in excellent health, he had rented a furnished house in Boston last fall and had become seriously ill after occupying one of the rooms as a study. His physician had told him that his symptoms indicated arsenical poisoning, and, upon examination, the wall-paper and the covering of a divan in the room were found to be heavily charged with arsenic.

It was shown that some of the articles of furniture referred to in the course of the hearing had been bought at well-known stores in Boston. It does not appear in the reports published by the Boston papers that ex-Governor Long submitted any testimony beyond the statement that "the paper manufacturers were discontinuing the use of arsenic in their products." The evidence thus far laid before the Committee seems to prove conclusively that legislation is needed either to prevent the application of this poison to goods in common domestic use or to compel manufacturers and merchants to give fair warning to purchasers when the poisonous goods are sold.

